# Extrinsic intestinal denervation modulates tumor development in the small intestine of Apc^Min/+^ mice

**DOI:** 10.1186/s13046-015-0159-0

**Published:** 2015-04-29

**Authors:** Verena Liu, Alexandra Dietrich, Michael S Kasparek, Petra Benhaqi, Marlon R Schneider, Michael Schemann, Hendrik Seeliger, Martin E Kreis

**Affiliations:** Department of General-, Visceral- and Vascular Surgery, Charité University Medicine, Campus Benjamin Franklin, Hindenburgdamm 30, D-12000 Berlin, Germany; Department of Surgery, Ludwig-Maximilian´s University, Campus Grosshadern, Munich, Germany; Gene Center, LMU Munich, Munich, Germany; Human Biology, Technische Universität München, Freising, Germany

**Keywords:** Intestinal cancer, Vagus nerve, Visceral innervation

## Abstract

**Background:**

Innervation interacts with enteric immune responses. Chronic intestinal inflammation is associated with increased risk of colorectal cancer. We aimed to study potential extrinsic neuronal modulation of intestinal tumor development in a mouse model.

**Methods:**

Experiments were performed with male Apc^Min/+^ or wild type mice (4 weeks old, body weight approximately 20 g). Subgroups with subdiaphragmatic vagotomy (apcV/wtV), sympathetic denervation of the small intestine (apcS/wtS) or sham operated controls (apcC/wtC) were investigated (n = 6-14 per group). Three months after surgical manipulation, 10 cm of terminal ileum were excised, fixed for 48 h in 4% paraformaldehyde and all tumors were counted and their area determined in mm^2^ (mean ± standard error of the mean (SEM)). Whole mounts of the muscularis of terminal ileum and duodenum (internal positive control) were also stained for tyrosine hydroxylase to confirm successful sympathetic denervation.

**Results:**

Tumor count in Apc^Min/+^ mice was 62 ± 8 (apcC), 46 ± 11 (apcV) and 54 ± 8 (apcS) which was increased compared to wildtype controls with 4 ± 0.5 (wtC), 5 ± 0.5 (wtV) and 5 ± 0.6 (wtS; all p < 0.05). For Apc^Min/+^ groups, vagotomized animals showed a trend towards decreased tumor counts compared to sham operated Apc^Min/+^ controls while sympathetic denervation was similar to sham Apc^Min/+^. Area covered by tumors in Apc^Min/+^ mice was 55 ± 10 (apcC), 31 ± 8 (apcV) and 42 ± 8 (apcS) mm^2^, which was generally increased compared to wildtype controls with 7 ± 0.6 (wtC), 7 ± 0.4 (wtV) and 7 ± 0.6 (wtS) mm^2^ (all p < 0.05). In Apc^Min/+^ groups, tumor area was decreased in vagotomized animals compared to sham operated controls (p < 0.05) while sympathetically denervated mice showed a minor trend to decreased tumor area compared to controls.

**Conclusions:**

Extrinsic innervation of the small bowel is likely to modulate tumor development in Apc^Min/+^ mice. Interrupted vagal innervation, but not sympathetic denervation, seems to inhibit tumor growth.

## Introduction

The autonomic nervous system plays a key role for gut function under physiological conditions. It helps to maintain homeostasis by influencing secretion, peristalsis, and smooth muscle tone to name just a few tasks [1].

Under pathological conditions, the autonomic nervous system takes part in defense responses to toxins or pathogens. This was elegantly shown by Borovikova et al. who demonstrated that vagal stimulation has a beneficial effect on survival during sepsis which was simulated by exposure to systemic lipopolysaccharide endotoxin [2]. The molecular basis of this interaction between autonomic nervous system and immune system is the release of acetylcholine (ACh) from peripheral vagal nerve endings. ACh binds to nicotinic ACh receptors on macrophages which subsequently reduce their tumor necrosis factor -α (TNF-α) production and release [2]. This interaction was named cholinergic anti-inflammatory reflex and seems to be relevant not only during sepsis but also when intestinal inflammation is present during colitis [3] or postoperative ileus [4].

Acute inflammation *per se* is a pathological condition that may have detrimental consequences for the organism. Chronic inflammation, however, may trigger other pathologies, such as malignant transformation of epithelial cells during ulcerative colitis or transition from chronic pancreatitis to pancreatic cancers [5-8]. The question arises whether this malignant transformation occurs solely secondary to chronic inflammation or whether modulation by the autonomic nervous system may also play a role. Considering previous observations that tumor cells carry acetylcholine receptors, this seems to be a possible mechanism [9].

We, therefore, hypothesized that autonomic innervation to the intestine may modulate development of intestinal tumors. Our aim was to test this hypothesis in an established animal model of intestinal tumor development, the Apc^Min/+^ mouse [10].

## Methods

### Animals

Experiments were performed with male Apc^Min/+^ k/o mice (4 weeks old, body weight approximately 20 g). C57BL/6 wild type animals were also investigated for each subgroup. Presence of the Apc^Min/+^ knockout mutation was tested by RT-PCR (reverse transcriptase polymerase chain reaction) from a tail biopsy prior to experiments. Animal experiments were approved by the appropriate local committee (Regierung von Oberbayern).

In Apc^Min/+^ mice, small intestinal tumors develop due to a defect in the protein product of the Adenomatous polyposis coli (APC) gene caused by a point mutation in this gene. In consequence, in the multiple intestinal neoplasia (Min) mouse the Wnt/B-catenin signaling cascade promoting cell proliferation is overactive [11].

### Surgical procedures

**Figure 1:**
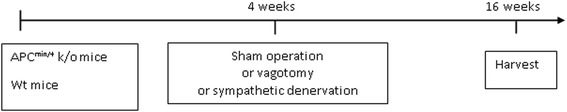
Experimental setup and timeline.

Separate subgroups underwent subdiaphragmatic vagotomy, surgical denervation at the level of the superior mesenteric artery or sham operation (laparotomy with surgical manipulation of the small intestine for 10 min; each n = 6–14, Figure 1).

Operations were performed following deep anesthesia with ketamine/xylazine (Park Davis, Berlin, Germany 100 mg/kg; Bayer, Leverkusen, 15 mg/kg intraperitoneally). For subdiaphragmatic vagotomy, the vagus nerve was isolated and cut at the level of the distal esophagus under microscopic vision (operating microscope, Wild M3Z, Heerburg, Switzerland). Small branches to the liver were identified and also divided. For denervation at the superior mesenteric artery, this vessel was isolated under microscopic vision from all surrounding tissue. Success of denervation here was confirmed by whole mounts of the muscularis of terminal ileum immediately proximal to the ileocolic junction and duodenum (internal positive control) which were obtainded at the time of final tissue harvest. Samples were stained for tyrosine hydroxylase (Merck Millipore, Merck Chemicals, Schwalbach) which is only expressed in sympathetic nerves within the enteric plexus. It is well established that vagal fibers course within the duodenal wall far into the distal small intestine. Surgical denervation at the level of the superior mesenteric artery, therefore, leads to complete sympathetic denervation of the small intestine, while parts of the vagal innervation along the intestinal wall remain intact. Successful sympathetic denervation of the small bowel was present when lack of immunohistochemical fluorescence for tyrosin hydroxylase in the ileum was observed, while it was still seen in tissue from the distal duodenum, which served as internal positive control.

### Tissue harvesting

**Figure 2:**
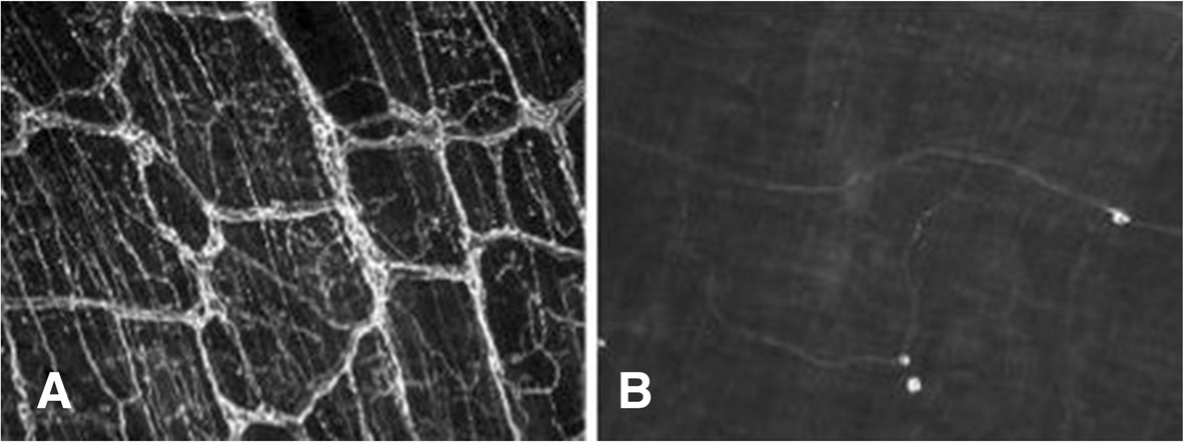
Representative images following denervation at the level of the superior mesenteric artery (SMA). **A**: Distal duodenum (internal positive control). **B**: Ileum following denervation at the SMA .This figure shows a representative whole mount preparation of terminal ileum from an animal that had undergone denervation at the level of the superior mesenteric artery (SMA). Stainings were performed for tyrosine hydroxylase which is present on sympathetic nerve fibers. Note the absence of tyrosine hydroxylase positive fibers in ileum of denervated animals showing lack of sympathetic innervation **(B)** compared to the normal distribution of sympathetic fibers in the distal duodenum which served as internal positive control **(A)**.

**Figure 3:**
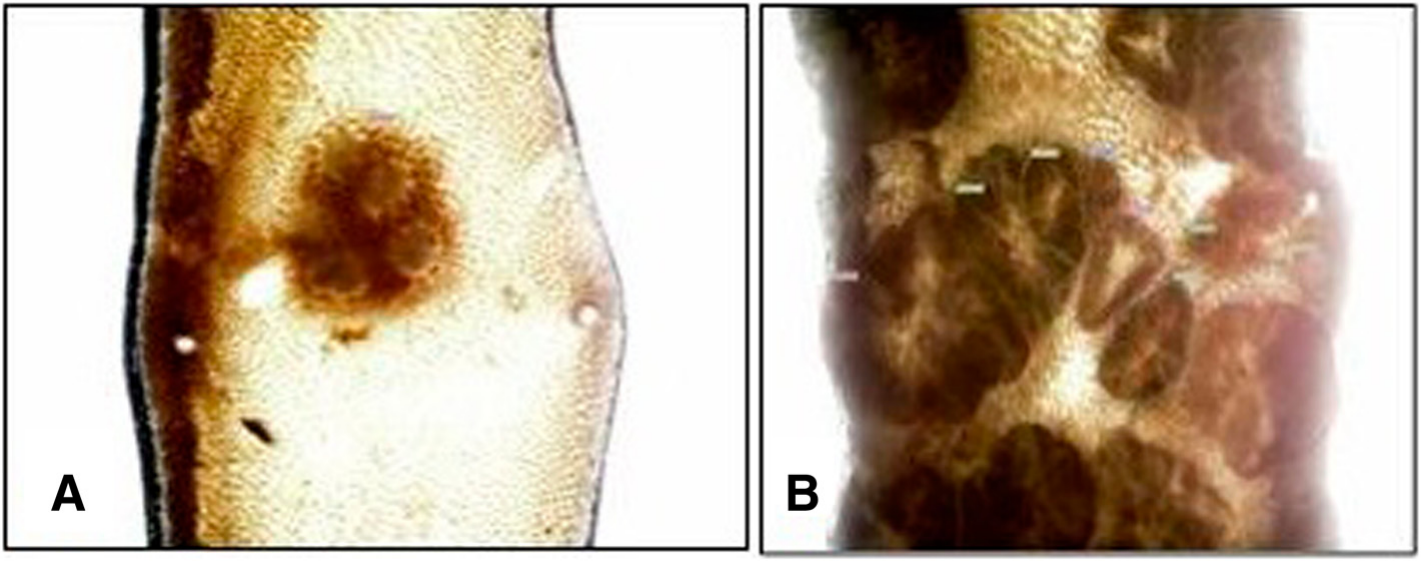
Representative images of adenoma growth in the small intestine. **A**: Single polyp in C57BL/6 (wt)-mice, **B**: Adenomas in Apc^Min/+^ mice. Representative images of adenomas in wild type **(A)** and Apc^Min/+^ animals **(B)**. Note the obvious difference in count and area of adenomas between both genotypes.

Three months after surgery, animals were sacrificed and a segment of 10 cm of terminal ileum beginning 1 cm proximal to the ileo-colic junction was excised in all subgroups. The segment was cut along the mesenteric border to expose the mucosa for evaluation of tumor numbers and area. Then it was fixed for 48 h in 4%-paraformaldehyde and the tumor number and area were evaluated (Figure 2). The distal duodenum was also harvested for staining with tyrosine hydroxylase (Merck Millipore, Merck Chemicals, Schwalbach) as a positive internal control in animals that had undergone denervation at the level of the superior mesenteric artery (Figure 3).

### Data analysis

All adenomas in the 10 cm segment of terminal ileum were counted manually with the help of a stereomicroscope (Olympus SZX7, Olympus GmbH, Hamburg, Germany). The area covered by adenomas along the 10 cm segment was determined by analysis of digital photographs taken under microscopic vision with the help of dedicated software. Descriptive statistics include mean ± SEM. Comparisons between the six subgroups were performed using ANOVA (one way analysis of variance). Post-hoc tests concerning comparisons between subgroups were performed using the Dunnetts test. P-values < 0.05 were considered statistically significant. The statistical analysis was performed with SPSS 22.

## Results

**Figure 4:**
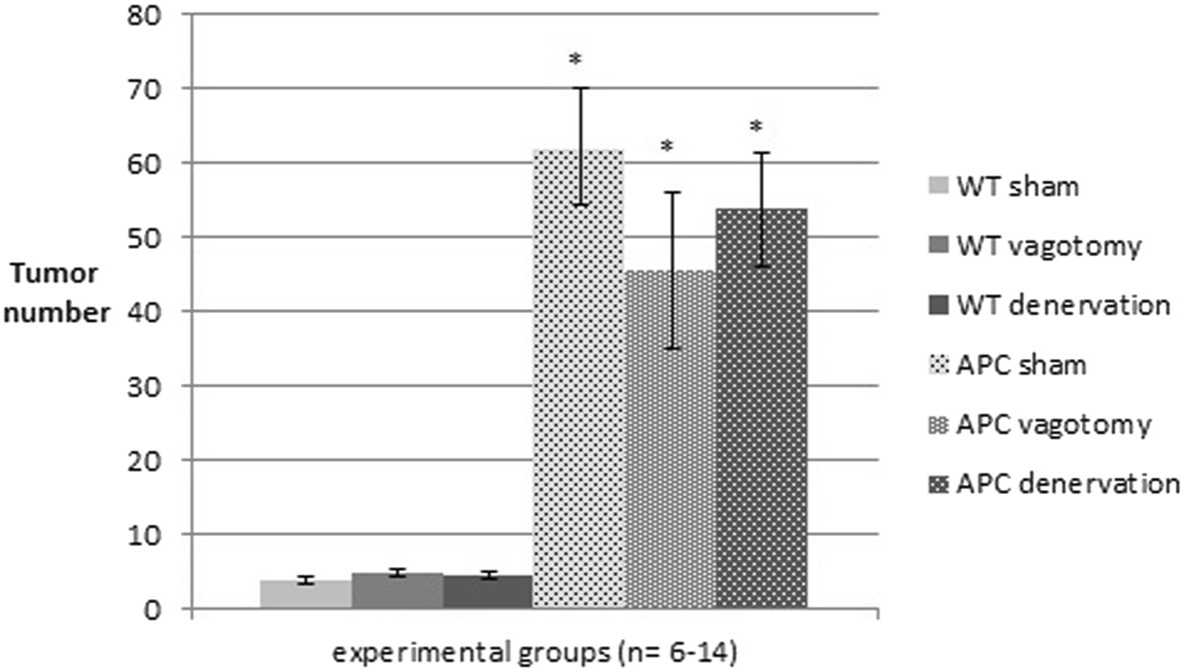
Ileal tumor count. Number of tumors in a 10 cm segment of terminal ileum in Apc^Min/+^ mice and wt animals after sham operation, vagotomy and complete mesenteric (sympathetic) denervation. Data are mean ± SEM. P < 0.05 for Apc^Min/+^ vs. wt (*).

A dramatic increase of tumor growth was observed in the terminal ileum of Apc^Min/+^ mice compared to wildtype control animals (Figure 4). In sham operated Apc^Min/+^ animals 62 ± 8 tumors were counted in the evaluated 10 cm segment of terminal ileum which was increased compared to 4 ± 0.5 tumors in wildtype controls (p < 0.05; Figure 4). Following subdiaphragmatic vagotomy, tumor count was reduced to 46 ± 11 in APC^min/+^ mice while tumor count following denervation at the level of the superior mesenteric artery was 54 ± 8 which was not significantly different compared to sham operated Apc^Min/+^ animals. Tumor counts were 5 ± 0.6 in wildtype controls following subdiaphragmatic vagotomy and 5 ± 0.5 following denervation at the mesenteric artery.

**Figure 5:**
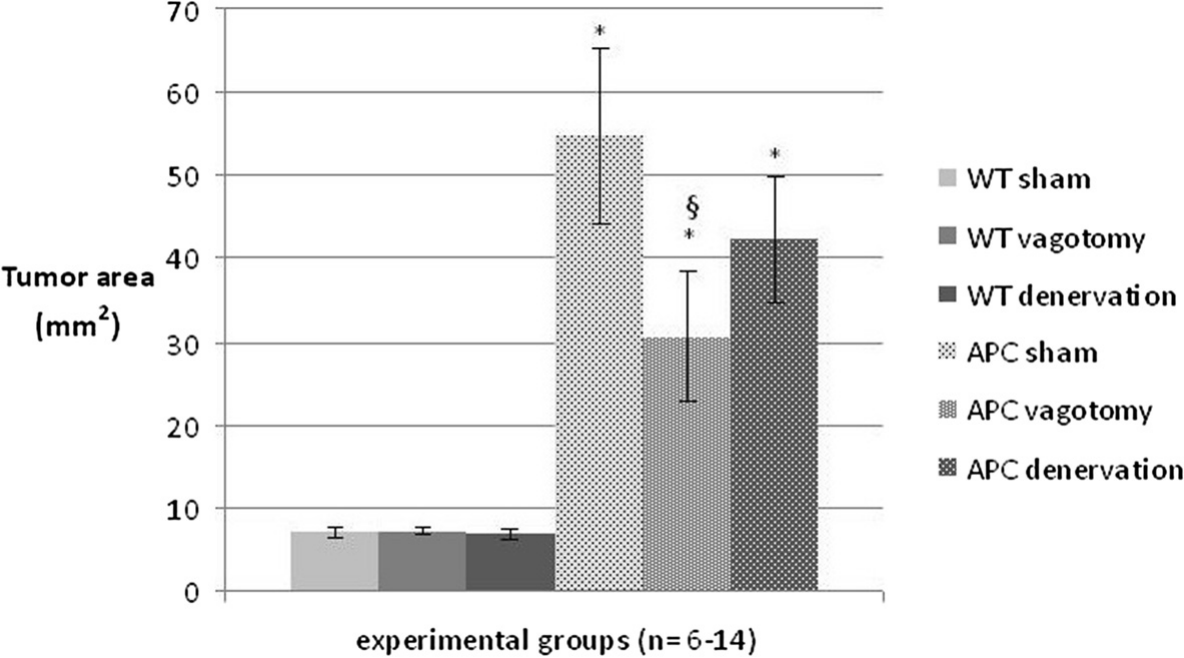
Area covered by tumor in the terminal ileum. Tumor area was determined in a 10 cm segment of terminal ileum in Apc^Min/+^ mice and wt animals after sham operation, vagotomy and complete mesenteric denervation. Data are mean ± SEM. P < 0.05 for Apc^Min/+^ vs. wt (*) and for APC sham vs. APC vagotomy (§).

The area covered by tumors in the 10 cm segment of terminal ileum in wildtype controls was 7 ± 0.6 mm^2^ following sham operation, 7 ± 0.4 mm^2^ after subdiaphragmatic vagotomy, and 7 ± 0.6 mm^2^ in animals having undergone denervation at the level of the superior mesenteric artery (Figure 5). The tumor area was increased in Apc^Min/+^ mice compared to corresponding wild type animals (all p < 0.05). In Apc^Min/+^ mice, corresponding areas covered by tumors were 54 ± 11 mm^2^ following sham operation, 30 ± 8 mm^2^ after subdiaphragmatic vagotomy, and 42 ± 8 mm^2^ following denervation at the superior mesenteric artery. In Apc^Min/+^ groups, tumor area was decreased in vagotomized animals compared to sham controls (p < 0.05), while mice denervated at the superior mesenteric artery were not different but showed a minor trend to decreased tumor area compared to sham operated controls.

## Discussion

In the present study, we observed a decrease in adenoma growth in Apc^Min/+^ animals that underwent previous subdiaphragmatic vagotomy compared to sham operated controls. Dissection of autonomic nerve fibers at the level of the superior mesenteric artery, which eliminates the sympathetic innervation to the small intestine and leaves the vagal innervation along the intestinal wall intact, had only a minor effect on reduction of adenoma growth.

This is the first time that a reduction of tumor growth following vagal denervation is described in an *in vivo* model of intestinal cancer. Interestingly, this effect was restricted to vagal denervation as it was not seen after sympathetic denervation at the level of the origin of the superior mesenteric artery.

Complete vagal denervation of the small intestine was ensured by cutting the ventral and dorsal trunk at the level of the diaphragm in addition to hepatic branches. This was done since the vagus nerve enters the abdomen as a ventral and dorsal trunk on the esophagus. The ventral trunk then divides into hepatic, ventral gastric and accessory celiac branches and the dorsal trunk in celiac and dorsal gastric branches [12]. Their branches descend on the celiac artery to near the celiac ganglion and then distribute to the small and large intestine along the mesenteric artery and its offshoots [1]. Therefore, complete vagotomy as performed in the present study at the level of the dorsal and ventral trunks including hepatic branches will eliminate vagal innervation of the stomach, duodenum and small intestine.

Denervation at the level of the superior mesenteric artery, in which all nervous tissue is stripped from the mesenteric artery, will destroy celiac vagal branches which deliver the majority of vagal efferents to the small intestine. This denervation, however, will leave the hepatic vagal branch intact, which was shown to also contribute to vagal innervation of the small intestine with fibers running within the intestinal wall [13]. Denervation at the level of the superior mesenteric artery, therefore, would leave part of the vagal innervation to the small intestine intact. This explains why a similar decrease in tumor numbers and area was not observed after mesenteric denervation at the superior mesenteric artery compared to subdiaphragmatic vagotomy. However, sympathetic denervation of the small intestine was complete following surgical denervation at the superior mesenteric artery as stainings for tyrosine hydroxylase were negative in these animals compared to internal positive controls. Thus, the sympathetic innervation appears to have no infuence on tumor growth in this model.

The main neurotransmitter of the efferent vagus nerve endings is acetylcholine. ACh has been characterized as an ubiquitous signaling molecule in neuronal as well as non-neuronal tissues with significant influence on tumor development. ACh activates nicotinic ligand gated ion channel receptors as well as G-protein coupled muscarinic receptors [14]. Several mechanisms through which ACh influences carcinogenesis and tumor progression were identified so far and may underlie our observations. This includes immunomodulatory function of ACh and its potential role in chronic inflammation and thus cancer development, where ACh might promote tumor growth through its anti-inflammatory and immunosuppressive actions [15,16]. ACh also has a proliferative effect on cancer cells [17] and may play a role as an important regulator in colorectal cancer. Signaling via the muscarinic receptor M3 (M3R), which is overexpressed in colon cancer, was found to have tumor promoting effects[18], as does signaling via the α7 nicotinic acetylcholine receptor (nAChR)[19].

The cholinergic anti-inflammatory pathway is a counter–regulatory pathway of the autonomic nervous system that contains pro- inflammatory responses. The vagus nerve has been identified as the main conduit and bilateral subdiaphragmatic truncal vagotomy can suppress this effect [20]*.* ACh can modulate macrophage function via the α7 subunit of the nicotinic acetylcholine receptor [21], thereby inhibiting the synthesis and release of the pro-inflammatory cytokines TNFα, interleukin −1 (IL-1), interleukin −18 (IL-18) and interleukin-6 (IL-6) [22]. On a molecular level, activation of the α7 subunit of the nicotinic acetylcholine receptor leads to inhibition of nuclear factor kappa B (NFκB), which is a well-studied downstream component of the mitogen activated protein kinases (MAPK) and regulates cell proliferation as well as synthesis of pro-inflammatory cytokines [3]. NFκB is prevented from translocating into the nucleus, either through inhibiting phosphorylation of the inhibitory protein IκBα or by activating the Jak (januskinase) 2-STAT (Signal Transducers and Activators of Transcription) 3 anti-inflammatory signaling pathway [23]. Following vagotomy the attenuating vagal effect is absent with subsequently increased cytokine levels and activation of the immune system [24]. This increased activation is likely to enhance immune surveillance with the potential consequence of decreased tumor growth that would explain reduced tumor count and surface area following vagotomy in our study.

Alternatively, ACh may have stimulated muscarinic receptors. Effects on muscarinic receptors include stimulation of carcinogenesis of colon carcinoma both *in vitro* and *in vivo*. Muscarinic receptors are expressed ubiquitously throughout the body and are involved in numerous physiologic processes. They are members of the large family of G-protein coupled receptors (GPCR). Five receptor subtypes M1-5 have been identified [18]. The receptors primarily expressed in colon cancer are the Gq-coupled M3 receptors (M3R). Overexpression of M3R has been found both in human colon cancer specimens [25] as well as in several colon cancer cell lines [9]. In addition to neuronal sources from autonomic nerve fibers, ACh is produced nonneuronally in colon cancer cells themselves [26]. The proliferative action of M3R depends in part on the transactivation of epidermal growth factor receptors (EGFR), resulting in intracellular signaling via both the MEK/ERK (mitogen-activated protein kinase kinase/extracellular signal regulated kinase) and PI3K/AKT (phosphoinositid-3-kinase/AKT) signaling pathways [27,28]. In the classical Gq pathway, phospholipase C (PLC) is known to stimulate two signaling events, the activation of proteinkinase C (PKC) and the release of calcium from intracellular stores. The transient mobilization of intracellular Ca^2+^ is followed by a sustained Ca^2+^ influx from the extracellular space. Both pathways converge on extracellular signal-regulated kinases 1 and 2 (ERK1/2) activation [14]. ERK1/2 belongs to the group of mitogen-activated protein kinases, which are involved in various cellular functions, such as cell growth, proliferation, differentiation and apoptosis [29]. The combined effects of EGFR and PKC inhibition are additive, suggesting that the EGFR and PKC pathways act in parallel to link M3R stimulation to ERK1/2 activation [30]. In addition to this, it has been demonstrated that M3R agonists stimulate actin stress fiber formation and human colon cancer cell migration and invasion [31]. In vivo, Raufman et al. showed that M3R knockout mice were resistant to the development of colon tumors in the azoxymethane-induced colon neoplasia model, which confirmed the actual role of M3R in colon cancer [32]. In the APC^min/+^ mouse model, reducing M3R expression and activation attenuates intestinal epithelial cell proliferation and neoplasia [33]. Furthermore, tumorigenesis was shown to be inhibited via a M3R mechanism following vagotomy in a gastric cancer model [34]. This suggests for our study, that vagotomy with subsequently decreased ACh release may have inhibited M3R activation with ensuing decreased tumor growth in these animals.

Another possible target is the α7 subunit of the nicotinic acetylcholine receptor, which is also expressed in human colorectal carcinoma specimen [16] and cancer cell lines [35]. It was demonstrated that nicotine acting on the α7 nACHR has many tumor promoting functions [36]. ACh could influence tumor progression in a similar way. Activation of α7 nAChR through nicotine involves the transactivation of EGFR through intracellular and L-type voltage sensitive calcium channels as well as the activation of protein kinase C (PKC). This leads to downstream activation of the serine/threonine kinase raf-1, the mitogen-activated kinases ERK1 and ERK2 and the transcription factors FOS, JUN and MYC [19,36]. Nicotine has also been found to upregulate vascular endothelial growth factor receptor (VEGFR) [19] and to stimulate angiogenesis via the α7 nAChR [37].

Although several mechanism through which acetylcholine influences tumor growth have been described, the role of the vagus nerve and the autonomic nervous system might still be far more complex. Sympathetic denervation did not lead to a significant reduction in tumor growth in Apc^Min/+^ mice, so the mechanism seems to be confined to the vagus nerve, potentially to ACh. It was shown that colon cancer cells also have receptors for other neurotransmitters, such as dopamine and norepinephrine [15]. Little is known yet about the interactions between different neurotransmitters and cytokines in the development of colon tumors. The precise molecular mechanisms by which the autonomic nervous system modulates intestinal tumor growth, therefore, remain to be elucidated.

In conclusion, intact vagal innervation seems to support tumor development under the special condition of the Apc^Min/+^ mouse model and tumor growth was attenuated following chronic vagotomy. The mechanism underlying this observation remains to be explored. Contrary to the vagus nerve, sympathetic innervation of the small intestine appears to have no obvious influence on tumor development.
